# Diagnostic and Prognostic Significance of Methionine Uptake and Methionine Positron Emission Tomography Imaging in Gliomas

**DOI:** 10.3389/fonc.2017.00257

**Published:** 2017-11-01

**Authors:** Kamalakannan Palanichamy, Arnab Chakravarti

**Affiliations:** ^1^Department of Radiation Oncology, The Ohio State University College of Medicine and Comprehensive Cancer Center, Columbus, OH, United States

**Keywords:** glioblastoma, positron emission tomography, methionine, radiation treatment planning, ^18^F-fluorodeoxyglucose, methylthioadenosine phosphorylase, metabolism

## Abstract

The present most common image diagnostic tracer in clinical practice for glioma is ^18^F-fluorodeoxyglucose (FDG) positron emission tomography (PET) for brain tumors diagnosis and prognosis. PET is a promising molecular imaging technique, which provides real-time information on the metabolic behavior of the tracer. The diffusive nature of glioblastoma (GBM) and heterogeneity often make the radiographic detection by FDG-PET inaccurate, and there is no gold standard. FDG-PET often leads to several controversies in making clinical decisions due to their uptake by normal surrounding tissues, and pose a challenge in delineating treatment-induced necrosis, edema, inflammation, and pseudoprogression. Thus, it is imperative to find new criteria independent of conventional morphological diagnosis to demarcate normal and tumor tissues. We have provided proof of concept studies for ^11^C methionine-PET (MET-PET) imaging of gliomas, along with prognostic and diagnostic significance. MET-PET is not widely used in the United States, though clinical trials from Japan and Germany suggesting the diagnostic ability of MET-PET imaging are superior to FDG-PET imaging for brain tumors. A major impediment is the availability of the onsite cyclotron and isotopic carbon chemistry facilities. In this article, we have provided the scientific rationale and advantages of the use of MET-PET as GBM tracers. We extend our discussion on the expected pitfalls of using MET-PET and ways to overcome them by incorporating a translational component of profiling gene status in the methionine metabolic pathway. This translational correlative component to the MET-PET clinical trials can lead to a better understanding of the existing controversies and can enhance our knowledge for future randomization of GBM patients based on their tumor gene signatures to achieve better prognosis and treatment outcome.

## Introduction

The complex nature of high-grade gliomas [glioblastoma (GBM)] makes radiologic detection inaccurate. It is essential to find new criteria either complementary to or independent of positron emission tomography FDG-PET diagnosis in distinguishing tumor and normal tissues. PET provides the real time metabolic monitoring of molecules and identifying molecules specific to cancer cells can play a crucial role in cancer diagnostics. Previously, through metabolic profiling of gliomas, we have shown that glioblastoma (GBM) cells and glioma stem cells (GSCs) accumulate methionine when compared to normal counter parts. Cellular uptake studies of methionine in GBM, GSC, and normal human astrocytes (NHAs) show a significant uptake of methionine by GBMs and GSCs when compared to NHA (1). This report provides the scientific foundation for ^11^C-Methionine-PET (MET-PET) imaging. In this article, we offer a rationale for incorporating MET-PET imaging in future large cohorts of brain tumor clinical trials and evaluate its prognostic value over conventional FDG-PET. Methionine uptake by GBM represents a metabolic abnormality that distinguishes cancerous from normal tissues. ^11^C-Methionine may be a better PET imaging agent than conventionally used 2-deoxy-2-^18^fluorodeoxyglucose (FDG) because the human body cannot biosynthesize methionine. Though there are a few studies reporting ^19^F-labeled amino acid and other analogs as tracers for PET imaging, FDG-PET is the widely used imaging modality for brain tumors. Therefore, we have made a comparison of FDG-PET vs. MET-PET in this article.

### Methionine Metabolic Regulation and Scientific Rationale from a Biological Perspective

Methylthioadenosine phosphorylase (*MTAP*) is a key enzyme essential for the methionine salvage pathway. *MTAP* catalyzes the cleavage of 5′-methylthioadenosine (MTA) into adenine and 5-methylthioribose-1-phosphate (MTR-1-P). Adenine is then used to generate AMP, whereas MTR-1-P is converted into methionine. In normal mammalian tissues, *MTAP* recycles purines and methionine consumed during polyamine synthesis. *MTAP* deficiency has been reported in both liquid and solid tumors, including gliomas (2). Previous studies have reported gliomas are frequently associated with abnormalities in chromosome 9 and the *MTAP* locus is located at 9p21.3. The shortest region of overlap of the deletions maps in the interval between the centromeric end of the interferon gene cluster and *MTAP* gene. This deletion is associated with high grade and recurrent gliomas suggesting that these alterations could contribute to the progression of GBM (3). Further, *MTAP* resides approximately 100 kb telomeric of *p16^INK4A^*. *MTAP* is usually codeleted with p16 (*cdkN2a/ARF*). Homozygous deletions of human chromosome 9p21 occur frequently in malignant cell lines and are common in primary gliomas.

In general, GBMs lack expression of the enzyme *MTAP*, due to either deletion or methylation of the *MTAP* promoter. Others, including us, have reported that in the majority of GBMs, *MTAP* is lost (1, 2). Copy number assays carried out on U87, U118, LN18, and LN229 GBM cell lines showed a value of “0” for U87, U118, and LN18 and “1” for LN229. These results coincide with the gene expression analysis and the production of MTA (1). MTA is secreted in the extracellular milieu of GBM cells, but not in NHAs. The availability of extracellular MTA suggests that GBM cells fail to recycle the methionine from MTA, resulting in its accumulation. *MTAP*, which recycles methionine, is lost in almost all cancers, resulting in an increased uptake of methionine. Therefore, MET-PET can efficiently delineate normal/tumor volume facilitating to a precise surgical resection and effective radiation treatment planning using higher radiation dose. ^11^C-MET uptake is lower in the normal brain and brain under inflammatory conditions when compared to ^18^F-FDG. Amino acid uptake is specific to cancer cells due to the deregulated amino acid transporters in contrast to normal cells. Therefore, cancers consume more methionine for extensive proliferation and survival, while normal cells do not. Inflammatory conditions occurring in GBM patients during and after therapies uptake FDG and tend to give false positive results, whereas this effect is not observed in MET-PET because the uptake of methionine is restricted to inflammatory tissues. This specificity for methionine qualifies that ^11^C-MET and other amino acid tracers are superior to ^18^F-FDG. Thus, it is important to incorporate MET-PET imaging in addition to MRI in GBM clinical trials, to create a more precise tumor volume delineation, which could spare normal brain tissues and lead to a better treatment outcome. Though brain tumor imaging using MET-PET has been reported three decades ago, however, it is still not practiced widely in clinics (4, 5).

### Methionine Reliance of Cancer Cells

The first report on methionine dependence in cancer cells was reported more than half a century ago by using rodent models bearing subcutaneous tumors from Walker-256 cells, in response to a diet devoid of methionine (6). Later, another investigation on transfer RNA methylation reported a metabolic defect in Walker-256 cells, supporting the methionine dependency of these carcinosarcoma cells (7). Further investigations on methionine dependency led to the discovery that malignant cells were unable to proliferate and survive in Met(−) Hcy(+) medium, whereas normal cells remain unaffected by the Met(−) Hcy(+) medium and could survive and grow (8). This concept has been validated by several subsequent studies, which have used many malignant cell lines from organs such as breast, bladder, colon, brain, kidney, skin, and prostate, and primary cells derived from fresh patient tumors are methionine dependent (9–11). The methionine dependency and its role in cancer growth control can be achieved using the methionine restriction strategy, particularly in cancers that require methionine for survival and proliferation (12). The deficiency of *MTAP* has been reported in both solid tumors and hematologic malignancies, specifically glioma (2), leukemia (13, 14), non-small cell lung cancer (15), hepatocellular carcinoma (16), gastric carcinoma (17), and melanoma (18). The aggressiveness of solid tumors has been linked with *MTAP* deficiency. Though there are challenges and advantages of targeting tumors which lack *MTAP* activity, *MTAP* deficiency in human GBM could be a potential target for tumor-specific chemotherapy (2, 19). In liquid tumors, cells lacking *MTAP* cannot metabolize MTA, and MTA is rapidly secreted to extracellular matrix (13). The metabolic discovery data from our study shows the expression of MTA in the extracellular compartment of GBM cell lines. MTA is excreted by GBM cell lines, but not by NHA. Additionally, *MTAP* rescue studies in xenografts suggest *MTAP* is a tumor suppressor (1). Collectively, all these studies suggest that methionine dependency can be exploited for therapeutic benefit and tracer diagnostics.

### Methionine As a Tracer for GBM

Methionine is an essential neutral amino acid that can readily cross the blood–brain barrier through neutral amino acid transporters and accumulates in an active tumor (20). The uptake of methionine in a normal brain is relatively low as compared to those with gliomas, hence providing a potential advantage over 2-deoxy-2-^18^fluorodeoxyglucose (^18^F-FDG) (21, 22). Our metabolic data shows that the accumulation of methionine is specific to GBM cells; therefore, it can be used as a tumor cell tracer in PET imaging. Previous studies have shown that methionine has higher specificity and sensitivity in PET imaging than ^18^F-FDG, which may be helpful for brain tumor detection, tumor delineation, and differential diagnosis of suspected glioma recurrence (23–31). In summary, methionine is a selective and sensitive tracer for cancer cells.

### Relevance of MET-PET Combined with Other Imaging Modalities

A head to head comparison of ^18^F-fluorodeoxyglucose (FDG) and ^11^C-methionine (MET) PET was made. FDG-PET portrayed malignant tumors as hot spots but was not able to delineate the extent of the tumor, whereas MET-PET, regardless of the degree of malignancy, outlined the tumors clearly as areas of increased ^11^C-methionine accumulation (32). Perfusion and diffusion MRI combined with MET-PET in low-grade gliomas (LGG), depicted hotspot regions, which corresponded with maximum tumor perfusion, and interestingly low diffusion in non-enhancing gliomas. MET-PET facilitated in identifying tumor regions with perfusion abnormalities before surgery, suggesting that pMRI/MET-PET could provide useful in treating LGG. This delineation is important in LGG for a better prognosis, avoiding surgical complications, and other undesirable effects (33). In high-grade gliomas, both MET-PET/CT and advanced MRI techniques demonstrated similar performance in identifying tumor recurrence posttherapy (34). Advanced MRI was more specific, whereas MET-PET/CT was more sensitive. Diagnostic performance of both modalities was almost the same in providing additional information on tumor biology, tumor growth kinetics, and new insights into the pathophysiology of malignant brain tumors. In LGG, a positive correlation between methionine uptake and proliferation, especially in astrocytic tumors, a correlation was noted between methionine accumulation and Ki-67 labeling index. The positive correlation of methionine uptake in low-grade brain tumors suggests that MET-PET can be used to estimate tumor malignancy in grade I–IV gliomas (35). Methionine uptake has been correlated with microvessel density in gliomas, and MET-PET has been used to identify patients responding to antiangiogenic therapies as well as for further monitoring and follow-up (36). All these reports suggest that MET-PET is a valuable tool in brain tumor prognosis.

### Advantages of MET-PET over FDG-PET

Methionine was reported as the more sensitive and selective radiotracer in evaluating tumor recurrence by comparing FDG-PET and MET-PET. In this cohort, 24 patients had tumor recurrence and 11 patients did not recur. On MET-PET findings 70% of cases were suggestive of recurrent tumors and 42% of FDG-PET findings. Further interobserver agreement for MET was 0.93 and 0.23 for FDG (37). The limitations of FDG-PET are as follows. FDG-PET imaging was based on the principle that malignant tumors are associated with increased glucose uptake, due to glycolysis. FDG is transported into the cell by glucose transporters, phosphorylated by hexokinase to form FDG-6 phosphate and accumulates within the cell. In LGG, due to lower glycolytic rates, FDG-PET sensitivity is low and tumor to normal brain contrast is challenging (38). The interobserver variability of MET-PET is minimal irrespective of the glioma grade, provides a straightforward visualization of tumorous accumulation, and is an easier technique to interpret. A study examining the ratios of methionine uptake of tumors: contralateral normal gray matter, along with metabolic tumor volume of MET-PET in 42 malignant glioma patients treated in the adjuvant setting, reported the potential of MET-PET in glioma management (39). Another study demonstrated the potential of combining MET-PET/MRI for assessing suspected primary brain tumors by comparing PET/MRI vs. MRI and reported a significant improvement in diagnostic confidence (40). A consecutive series of 109 glioma patients (NCT02518061) underwent preoperative MET-PET. Finding from MET-PET imaging significantly correlated with histological grade and IDH1 mutation status (41). Meta-analysis of brain tumors MET-PET has excellent diagnostic accuracy in differentiating brain tumors, whereas FDG-PET has limited diagnostic performance (42). FDG uptake is increased due to inflammation since macrophages and other inflammatory cells also have an increased glycolytic rate (43). Previous studies using mouse models bearing malignant tumors have demonstrated that methionine uptake was higher in viable cancer cells and was low in macrophages and other cellular components (44). In FDG-PET, the tumor-to-normal tissue boundary is obscure due to the uptake of FDG by gray matter. Under hypoxic conditions, FDG uptake is increased by both normal and cancer tissue, resulting in false positives. Due to the high glucose metabolism in normal brain, ^18^FDG is not a sensitive tracer in delineating tumor vs. normal tissue, whereas ^11^C-MET is a better tracer for imaging gliomas, because methionine transport is higher in tumors when compared to the insignificant amount of transport into the normal brain (45). Isotope ^11^C has a half-life of 20 min, whereas ^18^F half-life is 110 min. The relatively short half-life of ^11^C when compared to ^18^F means that the time available for tracer preparation and imaging is less and will require an onsite cyclotron. However, it is important to note that methionine fulfills the criteria of a brain tumor tracer since it can cross the blood–brain barrier. Additionally, MET-PET can be useful in the diagnosis of other types of cancers due to methionine uptake by cancer cells owing to the specific metabolic requirement.

### MET-PET and Radiation Treatment Planning

The prolific growth of sophisticated and accurate radiation therapy techniques, such as stereotactic radiotherapy, radiosurgery, intensity-modulated radiotherapy, proton therapy, etc., may aid to increase survival rates and quality of life. This increased survival benefit is possible because using high-dose irradiation on a limited target volume could eradicate tumor cells, while minimizing radiation exposure to adjacent normal tissues. To achieve this, current imaging modalities have to be improved to identify the clinical target volume (CTV)/gross target volume (GTV). We hope that this can be achieved through the integration of MET-PET into radiation treatment planning. Differentiating nonspecific postoperative changes in operated patients with GBM, and delineating gross tumor volume precisely, is important for radiation treatment planning. It has been shown that the sensitivity and specificity of MET-PET in differentiating non-tumoral tissue and LGGs are 78 and 87%, respectively (23, 46). In GBMs, previous reports have shown that MET uptake was located beyond gadolinium enhancement on MRI in 74% of cases. These findings have significant implications for the treatment strategy for GBM patients from both surgical resection and radiation treatment planning perspectives (24, 29, 30). In 31 pediatric high-grade gliomas, MET-PET delineates non-contrast enhancing tumors and identified regions having increased risk for recurrence and this prognostic power of MET-PET may lead to a better assessment for radiotherapy (47). The success of radiation therapy relies on the possibility of delivering high-dose irradiation accurately to a limited target volume. This will eradicate tumor cells, while minimizing radiation exposure to normal, functional adjacent tissue. Gross tumor volume delineation and new methods of defining the tumor volume more precisely will aid in advancing the radiation treatment planning. Therefore, new imaging modalities should be incorporated to accurately define the GTV and CTV. By assessing imaging on treatment volumes and clinical outcome, investigators report that MET-PET/CT has a significant impact on radiation therapy planning, and appears to be a predictor of clinical outcome in that group of patients (48). A study comparing the MET-PET and MRI imaging modalities in a trial of 39 patients with resected gliomas show that MET-PET has a greater specificity to outline the gross tumor volume with greater accuracy. They have also shown that sparing of normal brain tissue can be achieved by integrating MET-PET in tumor volume delineation (24). Among PET, MET-PET has a greater resolution (3–4 mm) than ^123^I-α-methyl tyrosine-single photon computed emission tomography (7 mm) and is more appropriate for use with high precision radiation techniques (49). A recent report shows that ^11^C-MET-PET has promising potential for precisely delineating target volumes in planning radiation therapy for postoperative patients with GBM (29). One of the limitations of ^11^C-MET-PET is the half-life time of about 20 min, and will require an onsite cyclotron, and may be limited to academic research centers.

### Key Metabolites in Methionine Pathway

**Figure 1:**
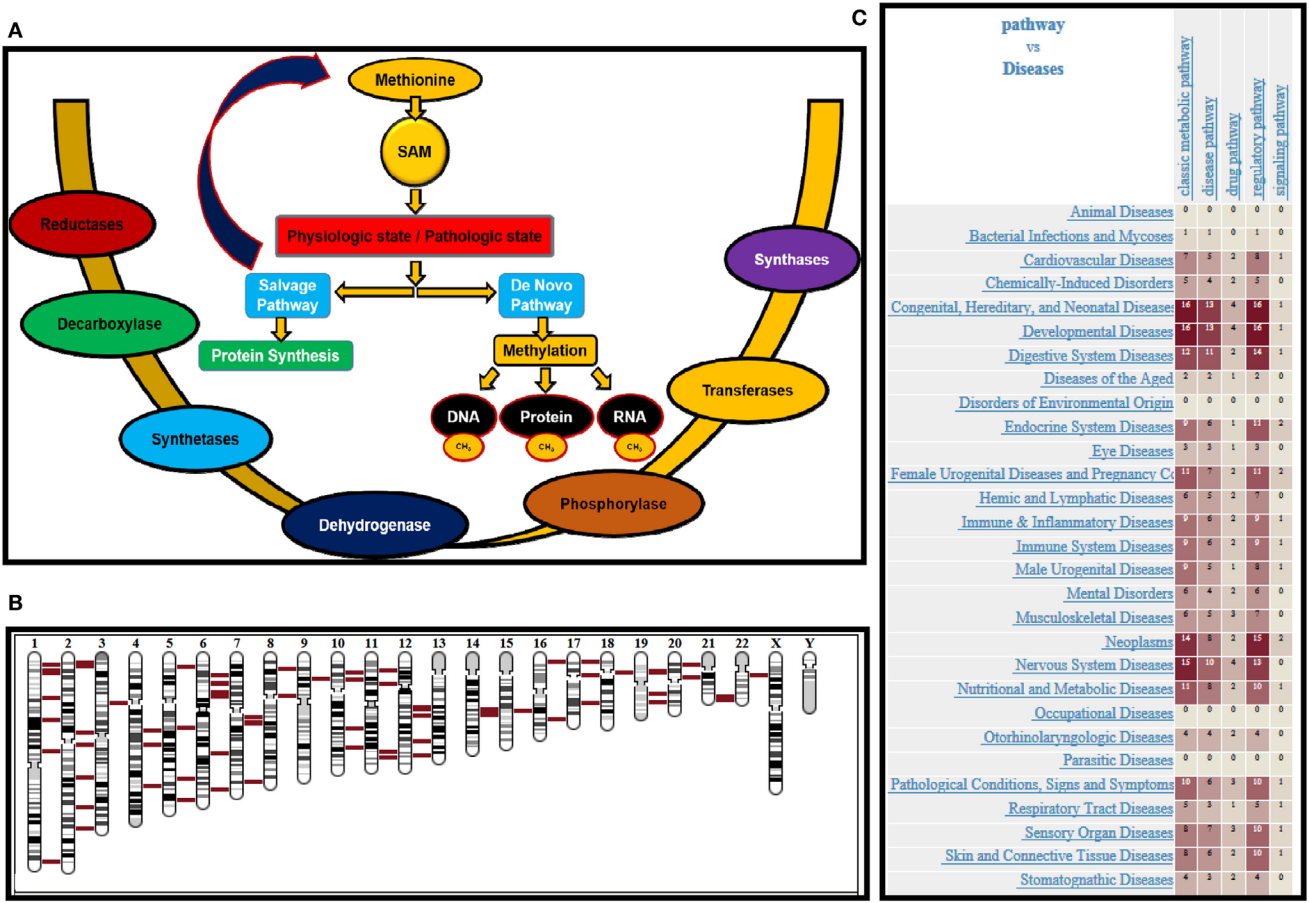
**(A)** Schematic representation of methionine uptake and utilization by nucleic acids and proteins under pathologic and physiologic conditions. Various enzymes governing the process are provided. **(B)** Chromosomal location of various enzymes involved in methionine regulation is shown in red. **(C)** Heat map provides the disease associations with genes that are mapped in methionine pathway.

Normal cells can survive and proliferate without methionine, while cancer cells would not due to the deregulation of various enzymes in methionine metabolic pathway. Exploiting this metabolic vulnerability could lead to better therapeutic intervention in cancers while sparing normal cells. Methionine can alter translational (protein), transcriptional (RNA), and epigenetic (DNA) status of cells. Methionine is the substrate for principle methyl donor S-adenosylmethionine (SAM) and variations in SAM levels can lead to hypo-/hypermethylation of DNA. Schematic methionine metabolic pathway diagram is shown in Figure 1A. Methionine is an essential amino acid that, when accumulated, is transformed into SAM, a key metabolite for polyamine synthesis. Methionine is portioned between protein synthesis and the *de novo* pathway, also referred to as methionine salvage pathway. In the salvage pathway methionine is converted to SAM, the principal methyl donor and is a substrate for nucleic acids (RNA and DNA) and proteins in biological systems. During methylation, SAM transfers methyl groups and is then converted to S-adenosylhomocysteine (SAH). SAH is further hydrolyzed to homocysteine (Hcy) then metabolized by methylation and trans-sulfuration salvaging methionine. The methionine metabolic pathway is deregulated in response to genetic or environmental stimuli, resulting in depletion of SAM or accumulation of SAH.

Biochemically after methionine uptake, methionine enters the methionine metabolic cycle. The initial members of the pathway methionine adenosyl transferase (*MAT1A*), and its paralog *MAT2A* catalyze the conversion of methionine to SAM, by recruiting ATP. SAM is the principle methyl donor in cells and regulates RNA, DNA, and protein methylation. SAM is utilized by both *de novo* and salvage pathways depending on the physiologic or pathologic state of cells. In the salvage pathway, SAM produces polyamines, while generating methylthioadenosine (MTA) as a byproduct. MTA is then phosphorylated by MTA phosphorylase (*MTAP*), producing 5′-methylthioribose-1-phosphate (MTR-1-P), which then salvage methionine as an end product. Conversely, in the *de novo* pathway, SAM methylates DNA, RNA, and protein regulated by methyl transferases. SAM, after donating methyl groups, is converted to SAH. SAH is then converted to homocysteine by adenosylhomocysteinase (*AHCY*), which further salvages methionine. Therefore, the patients should be stratified based on the status of enzymes *MAT 1A/2A, MTR1P, MTAP*, and *AHCY*. The chromosomal location of various genes regulating methionine pathway in the genomic context is shown in Figure 1B. All the chromosomes except “y” has genes that regulate methionine pathway, supporting the crucial role played by this essential amino acid. Further, the heat map (Figure 1C) shows the disease associations with genes mapped in methionine pathway. The data was generated using genome database (50).

### Correlative Markers for Successful MET-PET Clinical Trials

Though mounting evidence for using methionine as a PET tracer for successful diagnostic and treatment planning purpose, we should exercise caution and look for potential pitfalls. To identify the possible failure of MET-PET imaging in a subset of patients, we should equip ourselves with the activity status of key metabolites in methionine pathway. Methionine uptake will be determined primarily by the state of cells (cancer vs. normal). The sensitivity of MET-PET for gliomas including all grades has been estimated in the range 75–95%. Stratifying patients based on *MTAP* status could open the door for the potential utility of MET-PET as a diagnostic and prognostic tool for gliomas simultaneously addressing the sensitivity of MET-PET. Some of the key enzymes implicated in the methionine pathway are listed in Table 1 with their genomic location, nature, and function. Based on enzyme, methionine consumption/regeneration and/or SAM generation/consumption and/or SAH generation/consumption can occur. Any change in the status (hyperactive/hypoactive/inactive states) of enzymes due to the mutations that occur in these enzymes during cancer progression could potentially alter the methionine uptake. In cancer cells, enzymes regenerating methionine, enzymes consuming SAM, and enzymes generating SAH are crucial players in determining methionine uptake, since they can shift the equilibrium. Therefore, mutational or activity status of several of these enzymes listed in Table 1 should be used for correlative analysis. Based on this correlative analysis, eligibility criteria for patient stratification can be modified for a follow-up trial to achieve better treatment outcomes.

**Table 1:** Key enzymes in methionine metabolic pathway.

Gene symbol	Name	Chr	Start	Stop	Nature	Function
	**Transferase**					

*BHMT*	Betaine-homocysteine S-methyltransferase	5	79,111,781	79,132,290	Methionine regeneration	Conversion of betaine and homocysteine to dimethylglycine and methionine, respectively
*CARM1*	Coactivator-associated arginine methyltransferase 1	19	10,871,577	10,923,078	SAM consuming	Methylation of guanidino nitrogens of arginyl residues of proteins—acts specifically on histones and chromatin
*COMT*	Catechol-*O*-methyltransferase	22	19,941,740	19,969,975	SAM consuming	Transfers a methyl group from SAM to catecholamines, including the neurotransmitters dopamine, epinephrine, and norepinephrine
*DNMT1*	DNA methyltransferase 1	19	10,133,344	10,195,135	SAM consuming	Transfers methyl groups to cytosine nucleotides of genomic DNA—maintain methylation patterns
*DNMT3A*	DNA methyltransferase 3 alpha	2	25,232,961	25,342,590	SAM consuming	CpG methylation—preferentially methylate DNA linker between 2 nucleosomal cores and is inhibited by histone H1—repress transcription through the recruitment of HDAC activity
*DNMT3B*	DNA methyltransferase 3 beta	20	32,762,385	32,809,356	SAM consuming	Modulates dimethylation of promoter histone H3 at H3K4 and H3K9—function as a transcriptional corepressor by associating with ZHX1
*DOT1L*	DOT1-like histone lysine methyltransferase	19	2,163,963	2,232,578	SAM consuming	Methylates lysine-79 of histone H3. It is inactive against free core histones, but shows significant histone methyltransferase activity against nucleosomes
*GAMT*	Guanidinoacetate *N*-methyltransferase	19	1,397,026	1,401,570	SAM consuming	Converts guanidoacetate to creatine, using *S*-adenosylmethionine as the methyl donor
*GNMT*	Glycine *N*-methyltransferase	6	42,960,754	42,963,880	SAM consuming	Conversion of *S*-adenosyl-l-methionine (along with glycine) to *S*-adenosyl-l-homocysteine and sarcosine
*HNMT*	Histamine *N*-methyltransferase	2	137,964,068	138,016,364	SAM consuming	Histamine is metabolized by two major pathways: N(tau)-methylation *via* histamine *N*-methyltransferase and oxidative deamination *via* diamine oxidase
*KMT5A*	Lysine methyltransferase 5A	12	123,383,778	123,409,357	SAM consuming	Protein-lysine *N*-methyltransferase that can monomethylate Lys-20 of histone H4 to effect transcriptional repression of some genes
*MAT-1A/2A/2B*	Methionine adenosyltransferases	10,2,5	80,271,820	80,290,003	SAM generating	Catalyzes the transfer of the adenosyl moiety of ATP to methionine to form SAM and tripolyphosphate—SAM is the source of methyl groups for most biological methylations
*MRM2/FTSJ2*	Mitochondrial rRNA methyltransferase 2	7	2,234,291	2,242,198	SAM consuming	SAM-binding protein family—nucleolar protein and it may be involved in the processing and modification of rRNA
*MTFMT*	Mitochondrial methionyl-tRNA formyltransferase	15	65,001,512	65,029,639	SAM consuming	Formylates methionyl-tRNA in mitochondria. A single tRNA(Met) gene gives rise to both an initiator and an elongator species *via* an unknown mechanism
*MTR*	5-Methyltetrahydrofolate-homocysteine methyltransferase	1	236,794,304	236,903,981	Methionine regeneration	Catalyzes the transfer of a methyl group from methyl-cobalamin to homocysteine, yielding enzyme-bound cob(I)alamin and methionine—remethylates the cofactor using methyltetrahydrofolate
*NNMT*	Nicotinamide *N*-methyltransferase	11	114,295,813	114,312,516	SAM consuming	Catalyzes the N-methylation of nicotinamide and other pyridines to form pyridinium ions
*PCMT1*	Protein-l-isoaspartate (d-aspartate) *O*-methyltransferase	6	149,749,695	149,811,421	SAM consuming	Protein carboxyl methyltransferase—converts d-aspartyl and l-isoaspartyl residues resulting from spontaneous deamidation back to the normal l-aspartyl form
*PEMT*	Phosphatidylethanolamine *N*-methyltransferase	17	17,505,561	17,591,703	SAM consuming	Converts phosphatidylethanolamine to phosphatidylcholine by sequential methylation in the liver by utilizing SAM
*PNMT*	Phenylethanolamine N-methyltransferase	17	39,667,981	39,670,475	SAM consuming	Catalyzes the last step of the catecholamine biosynthesis pathway, which methylates norepinephrine to form epinephrine (adrenaline)
*PRMT1*	Protein arginine methyltransferase 1	19	49,676,166	49,688,450	SAM consuming	PRMTs methylate arginine residues on histones and other proteins by transferring methyl groups from SAM to terminal guanidino nitrogen atoms
*PRMT-2/-3/-5/-6/-7/-9*	Protein arginine methyltransferases	21,11,14,1,16, and 4			SAM consuming	PRMTs methylate arginine residues by transferring methyl groups from SAM
*RNMT*	RNA guanine-7 methyltransferase	18	13,726,647	13,764,556	SAM consuming	mRNA-capping methyltransferase—methylates the N7 position of the added guanosine to the 5-cap structure of mRNAs—binds RNA containing 5-terminal GpppC
*SETD7*	SET domain containing lysine methyltransferase 7	4	139,495,934	139,556,769	SAM consuming	SET domain containing lysine methyltransferase 7-lysine methyltransferases—transfers methyl groups from SAM to the lysine residues on histones, particularly histones H3 and H4
*SETDB1*	SET domain bifurcated 1	1	150,926,246	150,964,744	SAM consuming	Trimethylates Lys-9 of histone H3—epigenetic transcriptional repression by recruiting proteins to methylated histones
*SHMT1*	Serine hydroxymethyltransferase 1	17	18,327,860	18,363,563	Methionine regeneration	Serine hydroxymethyltransferase 1—interconversion of serine and glycine—this reaction provides one-carbon units for synthesis of methionine, thymidylate, and purines in the cytoplasm
*SMYD2*	SET and MYND domain containing 2	1	214,281,101	214,337,134	SAM consuming	Catalyze the transfer of methyl groups from S-adenosylmethionine (SAM) to the lysine residues on histones, particularly histones H3 and H4
*SUV39H1*	Suppressor of variegation 3–9 homolog 1	X	48,695,554	48,709,016	SAM consuming	N-terminal chromodomain and a C-terminal SET domain—catalyze the transfer of methyl groups from S-adenosylmethionine (SAM) to the lysine residues on histones, particularly histones H3 and H4

	**Reductase**					

*MSRB2*	Methionine sulfoxide reductase B2	10	23,095,498	23,122,013	Methionine regeneration	Reduces methionine sulfoxide back to methionine—methionine oxidation due to oxidative stress decreases the intracellular ROS
*MSRB3*	Methionine sulfoxide reductase B3	12	65,278,643	65,466,907	Methionine regeneration	Catalyzes the reduction of free and protein-bound methionine sulfoxide to methionine
*MTHFR*	Methylenetetrahydrofolate reductase	1	11,785,730	11,806,103	Methionine regeneration	Catalyzes the conversion of 5,10-methylenetetrahydrofolate to 5-methyltetrahydrofolate, a cosubstrate for homocysteine remethylation to methionine
*MTRR*	5-Methyltetrahydrofolate-homocysteine methyltransferase reductase	5	7,869,104	7,901,124	Methionine regeneration	Electron transferases involved in the reductive regeneration of cob(I)alamin (vitamin B12) cofactor required for the maintenance of methionine synthase in a functional state

	**Synthase**					

*CBS*	Cystathionine-beta-synthase	21	43,053,190	43,076,868	SAH consuming	Catalyze the conversion of homocysteine to cystathionine
*SMS*	Spermine synthase	X	21,940,573	21,994,837	Decarboxy-SAM consuming	spermidine/spermin synthase family-Catalyzes the production of spermine from spermidine and decarboxylated S-adenosylmethionine (dcSAM)
*SRM*	Spermidine synthase	1	11,054,592	11,060,053	Decarboxy-SAM consuming	

	**Decarboxylase**					

*AMD1*	Adenosylmethionine decarboxylase 1	6	110,814,621	110,895,713	SAM consuming	Important intermediate enzyme in polyamine biosynthesis

	**Synthetase**					

*MARS*	Methionyl-tRNA synthetase	12	57,487,953	57,516,655	Methionine consuming	Aminoacyl-tRNA synthetases—play a critical role in protein biosynthesis by charging tRNAs with their cognate amino acids

	**Phosphorylase**					

*MTAP*	Methylthioadenosine phosphorylase	9	21,802,636	21,865,971	SAM consuming	Salvage of both adenine and methionine—catalyzes the reversible phosphorylation of MTA to adenine and 5-MTR-1-P. Responsible for the first step in the methionine salvage pathway after MTA has been generated from S-adenosylmethionine

	**Miscellaneous**					

*AHCY*	Adenosylhomocysteinase	20	34,235,012	34,311,976	SAH generating	Catalyzes the reversible hydrolysis of SAH to adenosine and L-homocysteine—regulates intracellular SAH concentration
*CTH*	Cystathionine gamma-lyase	1	70,411,218	70,441,949	Methionine consuming	Encodes a cytoplasmic enzyme in the trans-sulfuration pathway that converts cystathione derived from methionine into cysteine. Glutathione synthesis in the liver is dependent upon the availability of cysteine
*EZH2*	Enhancer of zeste 2 polycomb repressive complex 2 subunit	7	148,807,372	148,884,662	SAM consuming	Polycomb-group (PcG) family—multimeric protein complexes involved in maintaining the transcriptional repressive state of genes over successive cell generations by methylation of histones

## Conclusion

We have provided the scientific rationale for initiating brain tumor clinical trials with MET-PET imaging for a better prognosis and treatment outcome. A prospective trial would be of great value, randomizing MET-PET and FDG-PET in a uniformly treated patient cohort with balanced *MTAP* status. In this study, key methionine, metabolic genes should be correlated with methionine uptake. Moving forward with MET-PET imaging trials, patients can be enrolled only in centers with the onsite cyclotron facility which appears to be a limitation. Nevertheless, we anticipate that this trial could change the diagnostic evaluation of gliomas and improve the care for the patients.

## Author Contributions

KP wrote the manuscript and AC edited the manuscript.

## Conflict of Interest Statement

The authors declare that the research was conducted in the absence of any commercial or financial relationships that could be construed as a potential conflict of interest.

## References

[1] Palanichamy K Thirumoorthy K Kanji S Gordon N Singh R Jacob JR Methionine and kynurenine activate oncogenic kinases in glioblastoma, and methionine deprivation compromises proliferation. Clin Cancer Res (2016) 22:3513–23. 10.1158/1078-0432.CCR-15-2308 26936918 PMC4947420

[2] Nobori T Karras JG Della Ragione F Waltz TA Chen PP Carson DA. Absence of methylthioadenosine phosphorylase in human gliomas. Cancer Res (1991) 51:3193–7. 1904005

[3] Olopade OI Jenkins RB Ransom DT Malik K Pomykala H Nobori T Molecular analysis of deletions of the short arm of chromosome 9 in human gliomas. Cancer Res (1992) 52(9):2523–9. 1568221

[4] Ericson K Lilja A Bergström M Collins VP Eriksson L Ehrin E Positron emission tomography with ([11C]methyl)-l-methionine, [11C]D-glucose, and [68Ga]EDTA in supratentorial tumors. J Comput Assist Tomogr (1985) 9(4):683–9. 3926834 10.1097/00004728-198507010-00005

[5] Lilja A Bergström K Hartvig P Spännare B Halldin C Lundqvist H Dynamic study of supratentorial gliomas with l-methyl-11C-methionine and positron emission tomography. AJNR Am J Neuroradiol (1985) 6(4):505–14. 2992257 PMC8335183

[6] Sugimura T Birnbaum SM Winitz M Greenstein JP Quantitative nutritional studies with water-soluble, chemically defined diets. VIII. The forced feeding of diets each lacking in one essential amino acid. Arch Biochem Biophys (1959) 81:448–55. 10.1016/0003-9861(59)90225-5 13638009

[7] Buch L Streeter D Halpern RM Simon LN Stout MG Smith RA Inhibition of transfer ribonucleic acid methylase activity from several human tumors by nicotinamide and nicotinamide analogs. Biochemistry (1972) 11:393–7. 10.1021/bi00753a015 4258169

[8] Halpern BC Clark BR Hardy DN Halpern RM Smith RA. The effect of replacement of methionine by homocystine on survival of malignant and normal adult mammalian cells in culture. Proc Natl Acad Sci U S A (1974) 71:1133–6. 10.1073/pnas.71.4.1133 4524624 PMC388177

[9] Breillout F Antoine E Poupon MF. Methionine dependency of malignant tumors: a possible approach for therapy. J Natl Cancer Inst (1990) 82:1628–32. 10.1093/jnci/82.20.1628 2213904

[10] Guo HY Herrera H Groce A Hoffman RM. Expression of the biochemical defect of methionine dependence in fresh patient tumors in primary histoculture. Cancer Res (1993) 53:2479–83. 8495409

[11] Poirson-Bichat F Goncalves RA Miccoli L Dutrillaux B Poupon MF. Methionine depletion enhances the antitumoral efficacy of cytotoxic agents in drug-resistant human tumor xenografts. Clin Cancer Res (2000) 6:643–53. 10690550

[12] Cavuoto P Fenech MF. A review of methionine dependency and the role of methionine restriction in cancer growth control and life-span extension. Cancer Treat Rev (2012) 38:726–36. 10.1016/j.ctrv.2012.01.004 22342103

[13] Kamatani N Carson DA. Abnormal regulation of methylthioadenosine and polyamine metabolism in methylthioadenosine phosphorylase-deficient human leukemic cell lines. Cancer Res (1980) 40:4178–82. 6781742

[14] Kamatani N Yu AL Carson DA. Deficiency of methylthioadenosine phosphorylase in human leukemic cells in vivo. Blood (1982) 60:1387–91. 6814551

[15] Nobori T Szinai I Amox D Parker B Olopade OI Buchhagen DL Methylthioadenosine phosphorylase deficiency in human non-small cell lung cancers. Cancer Res (1993) 53:1098–101. 8382555

[16] Kirovski G Stevens AP Czech B Dettmer K Weiss TS Wild P Down-regulation of methylthioadenosine phosphorylase (MTAP) induces progression of hepatocellular carcinoma via accumulation of 5’-deoxy-5’-methylthioadenosine (MTA). Am J Pathol (2011) 178:1145–52. 10.1016/j.ajpath.2010.11.059 21356366 PMC3069916

[17] Kim J Kim MA Min SY Jee CD Lee HE Kim WH. Downregulation of methylthioadenosin phosphorylase by homozygous deletion in gastric carcinoma. Genes Chromosomes Cancer (2011) 50:421–33. 10.1002/gcc.20867 21412930

[18] Stevens AP Spangler B Wallner S Kreutz M Dettmer K Oefner PJ Direct and tumor microenvironment mediated influences of 5’-deoxy-5’-(methylthio)adenosine on tumor progression of malignant melanoma. J Cell Biochem (2009) 106:210–9. 10.1002/jcb.21984 19097084

[19] Bertino JR Waud WR Parker WB Lubin M. Targeting tumors that lack methylthioadenosine phosphorylase (MTAP) activity: current strategies. Cancer Biol Ther (2011) 11:627–32. 10.4161/cbt.11.7.14948 21301207 PMC3084968

[20] Miyake K Okada M Hatakeyama T Okauchi M Shindo A Kawanishi M Usefulness of L-[methyl-11c]methionine positron emission tomography in the treatment of idiopathic hypertrophic cranial pachymeningitis – case report. Neurol Med Chir (Tokyo) (2012) 52:765–9. 10.2176/nmc.52.765 23095274

[21] Okada Y Nihashi T Fujii M Kato K Okochi Y Ando Y Differentiation of newly diagnosed glioblastoma multiforme and intracranial diffuse large B-cell Lymphoma using (11)C-methionine and (18)F-FDG PET. Clin Nucl Med (2012) 37:843–9. 10.1097/RLU.0b013e318262af48 22889772

[22] Singhal T Narayanan TK Jacobs MP Bal C Mantil JC. 11C-methionine PET for grading and prognostication in gliomas: a comparison study with 18F-FDG PET and contrast enhancement on MRI. J Nucl Med (2012) 53:1709–15. 10.2967/jnumed.111.102533 23055534

[23] Herholz K Holzer T Bauer B Schroder R Voges J Ernestus RI 11C-methionine PET for differential diagnosis of low-grade gliomas. Neurology (1998) 50:1316–22. 10.1212/WNL.50.5.1316 9595980

[24] Grosu AL Weber WA Riedel E Jeremic B Nieder C Franz M L-(methyl-11C) methionine positron emission tomography for target delineation in resected high-grade gliomas before radiotherapy. Int J Radiat Oncol Biol Phys (2005) 63:64–74. 10.1016/j.ijrobp.2005.01.045 16111573

[25] Kawai N Maeda Y Kudomi N Miyake K Okada M Yamamoto Y Correlation of biological aggressiveness assessed by 11C-methionine PET and hypoxic burden assessed by 18F-fluoromisonidazole PET in newly diagnosed glioblastoma. Eur J Nucl Med Mol Imaging (2011) 38:441–50. 10.1007/s00259-010-1645-4 21072512

[26] Aki T Nakayama N Yonezawa S Takenaka S Miwa K Asano Y Evaluation of brain tumors using dynamic 11C-methionine-PET. J Neurooncol (2012) 109:115–22. 10.1007/s11060-012-0873-9 22528799

[27] Arbizu J Tejada S Marti-Climent JM Diez-Valle R Prieto E Quincoces G Quantitative volumetric analysis of gliomas with sequential MRI and (1)(1)C-methionine PET assessment: patterns of integration in therapy planning. Eur J Nucl Med Mol Imaging (2012) 39:771–81. 10.1007/s00259-011-2049-9 22258713

[28] Galldiks N Dunkl V Kracht LW Vollmar S Jacobs AH Fink GR Volumetry of [(1)(1)C]-methionine positron emission tomographic uptake as a prognostic marker before treatment of patients with malignant glioma. Mol Imaging (2012) 11:516–27. 10.2310/7290.2012.00022 23084252

[29] Matsuo M Miwa K Tanaka O Shinoda J Nishibori H Tsuge Y Impact of [11C]methionine positron emission tomography for target definition of glioblastoma multiforme in radiation therapy planning. Int J Radiat Oncol Biol Phys (2012) 82:83–9. 10.1016/j.ijrobp.2010.09.020 21095072

[30] Miwa K Matsuo M Shinoda J Aki T Yonezawa S Ito T Clinical value of [(1)(1)C]methionine PET for stereotactic radiation therapy with intensity modulated radiation therapy to metastatic brain tumors. Int J Radiat Oncol Biol Phys (2012) 84:1139–44. 10.1016/j.ijrobp.2012.02.032 22520479

[31] Glaudemans AW Enting RH Heesters MA Dierckx RA Van Rheenen RW Walenkamp AM Value of 11C-methionine PET in imaging brain tumours and metastases. Eur J Nucl Med Mol Imaging (2013) 40:615–35. 10.1007/s00259-012-2295-5 23232505

[32] Ogawa T Inugami A Hatazawa J Kanno I Murakami M Yasui N Clinical positron emission tomography for brain tumors: comparison of fludeoxyglucose F 18 and L-methyl-11C-methionine. AJNR Am J Neuroradiol (1996) 17:345–53. 8938309 PMC8338383

[33] Berntsson SG Falk A Savitcheva I Godau A Zetterling M Hesselager G Perfusion and diffusion MRI combined with (1)(1)C-methionine PET in the preoperative evaluation of suspected adult low-grade gliomas. J Neurooncol (2013) 114:241–9. 10.1007/s11060-013-1178-3 23771511 PMC3742413

[34] D’Souza MM Sharma R Jaimini A Panwar P Saw S Kaur P 11C-MET PET/CT and advanced MRI in the evaluation of tumor recurrence in high-grade gliomas. Clin Nucl Med (2014) 39:791–8. 10.1097/RLU.0000000000000532 25036022

[35] Kato T Shinoda J Oka N Miwa K Nakayama N Yano H Analysis of 11C-methionine uptake in low-grade gliomas and correlation with proliferative activity. AJNR Am J Neuroradiol (2008) 29:1867–71. 10.3174/ajnr.A1242 18687745 PMC8118949

[36] Kracht LW Friese M Herholz K Schroeder R Bauer B Jacobs A Methyl-[11C]-l-methionine uptake as measured by positron emission tomography correlates to microvessel density in patients with glioma. Eur J Nucl Med Mol Imaging (2003) 30:868–73. 10.1007/s00259-003-1148-7 12692687

[37] Tripathi M Sharma R Varshney R Jaimini A Jain J Souza MM Comparison of F-18 FDG and C-11 methionine PET/CT for the evaluation of recurrent primary brain tumors. Clin Nucl Med (2012) 37:158–63. 10.1097/RLU.0b013e318238f51a 22228339

[38] Benard F Romsa J Hustinx R. Imaging gliomas with positron emission tomography and single-photon emission computed tomography. Semin Nucl Med (2003) 33:148–62. 10.1053/snuc.2003.127304 12756647

[39] Jung TY Min JJ Bom HS Jung S Kim IY Lim SH Prognostic value of post-treatment metabolic tumor volume from 11C-methionine PET/CT in recurrent malignant glioma. Neurosurg Rev (2017) 40:223–9. 10.1007/s10143-016-0748-1 27282449

[40] Deuschl C Goericke S Grueneisen J Sawicki LM Goebel J El Hindy N Simultaneous 11C-methionine positron emission tomography/magnetic resonance imaging of suspected primary brain tumors. PLoS One (2016) 11:e0167596. 10.1371/journal.pone.0167596 27907162 PMC5132315

[41] Lopci E Riva M Olivari L Raneri F Soffietti R Piccardo A Prognostic value of molecular and imaging biomarkers in patients with supratentorial glioma. Eur J Nucl Med Mol Imaging (2017) 44:1155–64. 10.1007/s00259-017-3618-3 28110346

[42] Zhao C Zhang Y Wang J. A meta-analysis on the diagnostic performance of (18)F-FDG and (11)C-methionine PET for differentiating brain tumors. AJNR Am J Neuroradiol (2014) 35:1058–65. 10.3174/ajnr.A3718 24029389 PMC7965151

[43] Nguyen QH Szeto E Mansberg R Mansberg V. Paravertebral infection (phlegmon) demonstrated by FDG dual-head coincidence imaging in a patient with multiple malignancies. Clin Nucl Med (2005) 30:241–3. 10.1097/01.rlu.0000156080.11877.b9 15764879

[44] Kubota R Kubota K Yamada S Tada M Takahashi T Iwata R Methionine uptake by tumor tissue: a microautoradiographic comparison with FDG. J Nucl Med (1995) 36:484–92. 7884515

[45] Jacobs AH Thomas A Kracht LW Li H Dittmar C Garlip G 18F-fluoro-L-thymidine and 11C-methylmethionine as markers of increased transport and proliferation in brain tumors. J Nucl Med (2005) 46:1948–58. 16330557

[46] Herholz K. Brain tumors: an update on clinical PET research in gliomas. Semin Nucl Med (2017) 47:5–17. 10.1053/j.semnuclmed.2016.09.004 27987557

[47] Lucas JT Jr Serrano N Kim H Li X Snyder SE Hwang S 11C-methionine positron emission tomography delineates non-contrast enhancing tumor regions at high risk for recurrence in pediatric high-grade glioma. J Neurooncol (2017) 132:163–70. 10.1007/s11060-016-2354-z 28078638 PMC8717215

[48] Schinkelshoek M Lopci E Clerici E Alongi F Mancosu P Rodari M Impact of 11C-methionine positron emission tomography/computed tomography on radiation therapy planning and prognosis in patients with primary brain tumors. Tumori (2014) 100:636–44. 10.1700/1778.19268 25688497

[49] Grosu AL Lachner R Wiedenmann N Stark S Thamm R Kneschaurek P Validation of a method for automatic image fusion (BrainLAB System) of CT data and 11C-methionine-PET data for stereotactic radiotherapy using a LINAC: first clinical experience. Int J Radiat Oncol Biol Phys (2003) 56:1450–63. 10.1016/S0360-3016(03)00279-7 12873691

[50] Shimoyama M De Pons J Hayman GT Laulederkind SJ Liu W Nigam R The rat genome database 2015: genomic, phenotypic and environmental variations and disease. Nucleic Acids Res (2015) 43:D743–50. 10.1093/nar/gku1026 25355511 PMC4383884

